# Pediatric Graves’ orbitopathy: TRAb and FT-3 are also prognostic factors in children—A tertiary center study

**DOI:** 10.1186/s13044-026-00286-7

**Published:** 2026-01-22

**Authors:** Karim Al-Ghazzawi, Michael Oeverhaus, Arber Rama, Cordula Kiewert, Nikolaos Bechrakis, Ying Chen, Inga Neumann, Anja Eckstein

**Affiliations:** 1 Department of Ophthalmology, University Hospital Essen, University of Duisburg-Essen, Hufelandstr. 55, 45147 Essen, Germany; 2 Ophthalmology Practice Dres. Oeverhaus, Rietberg, Germany; 3 Division of Pediatric Endocrinology, Department of Pediatrics II, Reference Centre Endo-ERN, University Hospital Essen, University of Duisburg-Essen, Essen, Germany

**Keywords:** Biomarker, Thyroid eye disease, GO, TED, Age, Graves’ orbitopathy, Strabism, Reoperation

## Abstract

**Purpose:**

Graves’ orbitopathy (GO) is an autoimmune disease of the orbit that occurs most often in relation to autoimmune. The clinical picture varies and is dependent on many risk factors, especially age, antibody levels and the quality of control of thyroid function. This study aimed to (1) compare the clinical characteristics of pediatric and adult Graves’ orbitopathy (GO), (2) identify factors associated with remission in pediatric GO, and (3) assess the influence of thyroid treatment modality on TRAb dynamics in pediatric patients.

**Methods:**

We reviewed the medical records of all pediatric patients with GO (< 18 years) and compared the results with those of a random sample of 482 (18–50 years old) adult patients from the Graves’ Orbitopathy Database (GODE), which includes 4260 patients from our tertiary referral center. A subcohort analysis of pediatric GO patients receiving definitive (surgical) thyroid or medical thyroid treatment was conducted. Risk stratification for remission in pediatric GO patients with the help of univariate as well as multiple logistic regression for different variables, including the serological laboratory results of Free Triiodothyronine (FT-3), Free Thyroxine (FT-4), TSH-receptor autoantibodies (TRAb) and antithyroglobulin antibodies (anti-TG), was conducted. Only those with complete data sets were included in the statistical analysis.

**Results:**

Clinical presentation varied significantly between pediatric and adult patients, with children showing mostly mild manifestations (81% vs. 48.7%, *p* < 0.0001), resulting in a much lower need for anti-inflammatory treatments and rehabilitative surgery. Univariate analyses revealed a significantly decreased probability of remission in nonthyroidectomized pediatric GO patients with high FT-3 or TRAb levels at the first clinical presentation (*p* < 0.05). The surgically managed pediatric GO cohort had higher initial TRAb levels but quicker TRAb normalization over time and higher serological remission rates (TRAb < 1,75UI) (38% vs 74%, respectively) than did the nonthyroidectomized pediatric subgroup at the last clinical presentation. Interestingly, pediatric GO patients in whom antithyroid drug (ATD) therapy was initiated only at or after their first clinical visit at our referral center presented greater clinical activity and prolonged TRAb normalization duration than patients who were already receiving anti-thyroid drugs did (*p* < 0.005).

**Discussion:**

FT-3 and TRAb levels are also prognostic factors for remission in pediatric patients with GO. International guidelines for the management of pediatric GO have divided opinions on the duration of anti-thyroid drugs prior to definitive surgical thyroid therapy (2–5 years). Our results highlight the prognostic role of serum biomarkers (FT-3, FT-4, TSH-receptor autoantibodies (TRAb)) at first clinical presentation and hint to positive effects of thyroidectomy in pediatric GO patients with high FT-3 or TRAb and therefore unlikely remission in the conservative treated subpopulation.

**Supplementary Information:**

The online version contains supplementary material available at 10.1186/s13044-026-00286-7.

## Introduction

Graves’ orbitopathy (GO)/thyroid eye disease (TED) is an autoimmune-mediated disorder, primarily associated with Graves’ disease (GD), that results in inflammation of the extraocular muscles and orbital connective tissue, leading to progressive eye dysfunction [1–4]. This condition is triggered by TSH receptor autoantibodies (TRAbs) that stimulate TSH receptors on orbital fibroblasts, which also leads to the costimulation of the growth factor IGF-1 receptor. In addition, CD40 expression on orbital fibroblasts attracts activated T cells, initiating an inflammatory cascade [5, 6]. Activated orbital fibroblasts release proinflammatory cytokines, produce hyaluronic acid, and differentiate into adipocytes and myofibroblasts [7–11]. Typically, GO incidence rates show a bimodal peak and occur in adults (40–49 years and 60–69 years) [1]. Since GD is much rarer in children than in adults, with an estimated incidence rate of 4.58 per 100,000 people per year, pediatric GO is also rare [12–14]. Pediatric Graves’ orbitopathy (GO) occurs in approximately one-third of pediatric GD cases, more commonly in children with a family history of autoimmune thyroid disease (up to 60%) [15] and in regions with higher smoking prevalence [16]. According to a European expert questionnaire [12, 16], 70% of professionals initially recommend a wait-and-see policy for pediatric GO, and intervention with steroids can be considered at progression in cases of worsening or still-active eye disease despite euthyroidism [12, 17]. Once children are diagnosed with GD, their risk of developing GO is similar to that of adults, with a comparable predominance in females. [16, 18] However, pediatric GO tends to be milder than adult GO, and it primarily induces proptosis. The mildness of GO in children remains an area of limited understanding. More severe manifestations, such as restrictive strabismus and compressive neuropathy, are generally rare in pediatric patients and are more prevalent in adult populations. The lower incidence of smoking in children (4% compared with 47% in adults) is believed to be one key factor in the milder clinical presentation of GO in pediatric patients [12]. The management of GO in pediatric patients typically follows a multidisciplinary approach. The first-line treatment for hyperthyroidism is antithyroid medication, with methimazole being the preferred agent because of its safety profile compared to propylthiouracil, which is associated with severe hepatotoxicity in children. For patients in whom remission is not achieved with medication, definitive treatments such as radioactive iodine (RAI) therapy or surgical thyroidectomy may be considered. In most GO patients, immunosuppressive treatment is not indicated since inflammatory signs are rare. Proptosis and lid retraction are followed by waiting and receiving thyrostatic treatment. The rare cases with swelling and motility impairment are treated with iv glucocorticoids (GC) [17]. For severe cases of GO later in the course of treatment, surgical interventions such as orbital decompression or eyelid surgery may be performed in those with significant proptosis or eyelid retraction, leading to exposure and appearance issues. Surgeries are typically delayed until the patient is in the static phase of GO to minimize risks and optimize outcomes. Surgical intervention in pediatric patients with GO remains rare. Owing to the rarity of GO in pediatric populations, most studies to date have been small, retrospective case series. The current study contributes the largest monocentric retrospective series to date, offering valuable insights into the demographics, clinical signs, and outcomes of GO and hyperthyroidism.

The aim of our retrospective study was to compare the clinical characteristics of pediatric patients with those of adult patients with GO. Follow-up data were studied to determine which risk factors contribute to low remission rates of TED (serological remission). Ionescu et al. conducted the largest multicentric retrospective study thus far (*n* = 115) [19]. The authors suggest that both pediatric and adult patients with GO present comparable clinical findings regarding both soft tissue involvement and proptosis; therefore, pediatric patients with GO can also benefit from surgical interventions. The objectives of this study were threefold: (1) to compare the clinical features of pediatric and adult GO at first presentation to a tertiary referral center, (2) to identify factors associated with remission in pediatric GO—including serological remission defined by TRAb normalization—and (3) to evaluate the effect of thyroid treatment modality (antithyroid drugs versus total thyroidectomy) on the trajectory of TRAb levels in pediatric patients.

## Patients and methods

### Study population

For this retrospective study, we analyzed medical records from pediatric GO patients who visited our EUGOGO (European Group On Graves’ Orbitopathy) tertiary referral center from January 2008 until December 2023. Baseline characteristics and the course of the disease (treatments, surgeries) were assessed from the patient charts. To compare these pediatric GO patients with adult patients, we used a subgroup (only 18–50 years) of our previously published cohort, which was a random sample of our Graves’ Orbitopathy Database Essen (GODE, *n* = 4260) [20].

### Thyroid management prior to consultation

In all cases, thyroidectomy refers to total thyroidectomy, as subtotal thyroidectomy is not performed at our institution, in accordance with current international guidelines.

### Thyroid medication at first clinical presentation

“Block” refers to standard antithyroid drug (ATD) monotherapy using a dose-titration regimen (typically methimazole). It does not include block-and-replace therapy. Patients categorized under ‘Block’ may have initiated ATD treatment shortly before first presentation and therefore had not yet reached a stable titration phase.

### Laboratory evaluation

The Elecsys Anti-TSH Receptor (TRAb) was measured via automated competitive binding immunoassays, namely, an electrochemiluminescent immunoassay (Roche Diagnostics, Mannheim, Germany) with a cutoff value of 1,75 IU/I, which is used for the clinical routine at the University Duisburg-Essen. The TRAb assay by Roche is a competitive binding immunoassay facilitating the production of M22 monoclonal antibodies, which bind to TSHR with high affinity.

Anti–thyroid peroxidase antibodies (anti-TPO) and anti-thyroglobulin antibodies (anti-Tg) were quantified using the Elecsys Anti-TPO and Elecsys Anti-Tg electrochemiluminescent immunoassays (Roche Diagnostics, Mannheim, Germany). These assays use a sandwich ECLIA format with biotinylated and ruthenium-labeled antigen complexes for high-specificity antibody detection. Manufacturer reference ranges: Anti-TPO: < 34 IU/mL, Anti-Tg: < 115 IU/mL

Serum thyroid parameters were assessed using standardized automated immunoassays routinely employed in the central laboratory of the University Hospital Essen. Free thyroxine (FT4) and free triiodothyronine (FT3) were measured using the Atellica IM Free Thyroxine and Atellica IM Free Triiodothyronine assays (Siemens Healthineers, Eschborn, Germany). These competitive chemiluminescent immunoassays use an acridinium ester–based detection system, in which endogenous FT4 or FT3 competes with labeled analyte for monoclonal antibody binding sites. Manufacturer reference ranges: FT4: 0.8–1.8 ng/dL (≈10–23 pmol/L) FT3: 2.3–4.2 pg/mL (≈3.5–6.5 pmol/L)

Thyroid-stimulating hormone (TSH) was measured using the Atellica IM TSH assay (Siemens Healthineers), a third-generation sandwich chemiluminescent immunoassay using monoclonal anti-TSH antibodies and acridinium ester detection. Manufacturer reference range: TSH: 0.4–4.0 mIU/L

### Clinical assessment

All patients were assessed by a team comprising specialized ophthalmologists (AE, MO, YC, IN) and skilled orthoptists. The diagnosis of GO was established by identifying typical clinical signs during the examination, which included measurements of Best Corrected Visual Acuity (BCVA), slit-lamp biomicroscopy, applanation tonometry, funduscopy, Hertel exophthalmometry, assessment of subjective diplopia, and objective measurement of deviation via the prism-cover test and measurement of monocular excursions [21]. In the absence of thyroid disease, we utilized clinical signs, MRI or CT images, and levels of thyroid-specific antibodies (TRAb, Anti-TPO) to diagnose euthyroid GO. The activity of GO was evaluated via the Clinical Activity Score (CAS) classification system [22, 23]. A CAS of ≥3/7 indicated active GO. Furthermore, we classified GO severity as mild, moderate-to-severe, or sight-threatening according to the EUGO criteria [21]. Motility dysfunction was defined as a decrease in the number of 360° explants below ≤ 330° in both cohorts. Diplopia was assessed as motility dysfunction in addition to deviance in the alternative prism cover test (APCT), with subjective double vision of the patient. Proptosis was positive if the Hertel difference in both eyes was ≥2 mm or if the Hertel exophthalmometry was ≥ 17 mm in pediatric patients and ≥ 18 mm in adult patients. In addition, we scored the soft tissue inflammation signs derived from CAS more gradually as follows: spontaneous retrobulbar pain (0–1), painful eye movement (0–1), upper lid edema (0–2), lower lid edema (0–2), conjunctival injection (0–1), chemosis (0–1), lid redness (0–1) and swelling of the caruncle or placenta (0–1). The sum was used to calculate the clinical soft tissue score (STS).

### Statistical evaluation

To analyze metric data, median values ($\widetilde {\bf{\it{x}}}$) and ranges or means and standard deviations (SDs) were computed. Student’s t test (two-tailed) was used to assess differences between groups if the D’Agostino-Pearson omnibus normality test indicated a normal distribution; otherwise, the Mann‒Whitney test was used. Fisher’s exact test was used to examine the group distributions of binary variables. A linear regression was performed for each variable on its own through an intercept/main effects model to determine the correlation between each variable and the independent dichotomous (remission) variable. Multivariable logistic regression analyses with the dependent variable (remission) as a dichotomous variable were performed with the models intercept as well as main effects for the pooled variables within the same models. A level of statistical significance was defined as two-tailed with 2α < 0.05. All calculations were performed via SPSS (IBM SPSS Statistics, Chicago, IL, USA, Version 22.0.0) and GraphPad Prism (Prism 9 for Windows, Software, Inc., San Diego, CA, USA, Version 9.0.0). *p* values are provided descriptively without α adjustment for multiple testing.

## Results

### Study population

The analysis included a total of 570 patients with GO. Group 1 was composed of 92 patients who were 18 years old or younger (mean age 12.7 ± 3 years, range 3.7–17.9), whereas Group 2 was composed of 482 adult patients (mean age 39.3 ± 8 years, range 19.3–49.9). Both groups were mainly composed of GO patients with autoimmune hyperthyroidism (89.1% in pediatric GO patients vs 82.7% in adult GO patients), *p* > 0.163. Table 1 summarizes the demographics of the study population.

**Table 1:** Patient characteristics in both cohorts

	< 18 (*n* = 86)	> 18–50 (*n* = 482)	p
Age at first clinical presentation	12.7±3	39.3±8	< 0.0001a
Median duration (months) of GO symptoms before presentation at our tertiary referral center	8.4	12.6	0.005b
consultation period (mean time in months from first till last consultation at our department)	16.2±21	11.7±17	0.0001b
**Symptoms at first clinical presentation**			
Proptosis	84%	80%	> 0.05b
Lid-retraction	29%	38%	0.09b
Soft Tissue inflammation (STS > 2)	42%	68%	< 0.0001b
Motility disfunction	22%	74%	< 0.0001b
Diplopia	0%	17%	< 0.002b
DON	1%	3%	> 0.05b
**Thyroid management prior to consultation**			
Total Thyroidectomy	10.8%	27.7%	0.0004b
RAI	3.2%	19.5%	< 0.0001b
**Thyroid medication at first clinical presentation**			
Block	44.2%	25%	0.0006b
Block and replace	25.5%	2%	< 0.0001b
L-Thyroxin monotherapy	18.6%	48.8%	< 0.0001b
No Medication	11.6%	23%	< 0.01b
**Therapies received till last show-up**			
Steroids	15.2%	41.4%	0.0006b
Orbital irradiation	0%	20%	< 0.0001b
Lid-surgery	3.2%	12%	< 0.001b
Eye muscle surgery	0%	13%	< 0.0001b
Orbital decompression	6.5%	20%	< 0.001b
**Thyroid status at last clinical presentation**			
Total Thyroidectomy	22.8%	34.4%	< 0.05b
RAI	5.4%	22%	0.0002b

Unless otherwise stated, the data are presented as the means ± SDs or proportions (%) or medians ($\tilde x)$[range]; a: t test/Mann‒Whitney test, b: Fisher’s exact test

### Patient characteristics

The mean duration from the first symptoms of Graves’ thyroid disease until the first ophthalmological examination at our tertiary referral center was significantly shorter in pediatric GO patients (8.4 vs. 12.6 months), *p* < 0.001; additionally, younger patients had significantly longer consultation periods (16.2±21 months, median 8.3 vs. 11.7 ± 17 months, median 3.7 months, *p* = 0.0001). While 43% of the adult patient cohort had already received corticosteroids prior to ophthalmological consultation, only 5/92 of the pediatric GO cohort had received corticosteroids.

## Clinical examination at first consultation

**Fig. 1:**
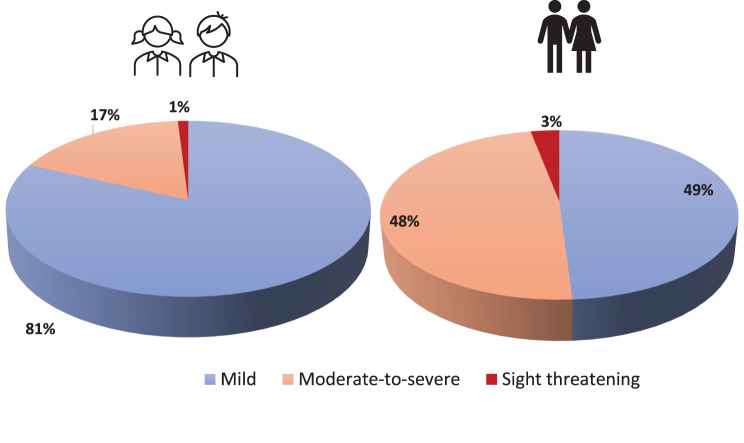
Severity of GO patients stratified by age groups (< 18 vs 18–50)

**Fig. 2:**
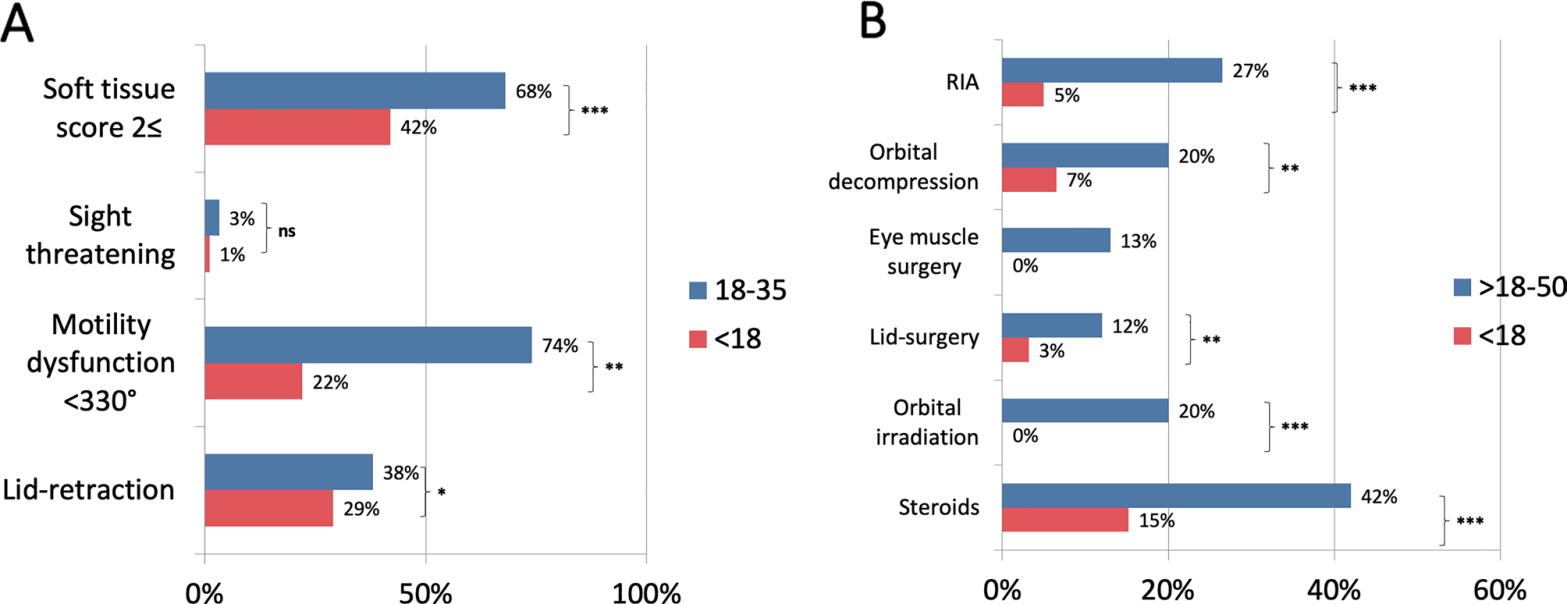
(**A**) appearance in GO patients stratified by age as proportions. (**B**) treatments for GO patients stratified by age as proportions. (Fisher’s exact test, * = 0.05, ** < 0.01, *** < 0.001)

Pediatric GO patients presented significantly more often with mild stages than did adult patients (81% vs. 48.7%, *p* < 0.0001) and less often with moderate-to-severe GO (17% vs 43.7%, *p* < 0.0001; Fig. 1). Sight-threatening manifestations occurred in 1 (1%) pediatric GO patient and in 15 (3%) adult GO patients. To reveal differences in the clinical appearance of the GO between pediatric and adult patients at the first clinical presentation, we compared the symptoms of both cohorts in the most “treatment-naive” picture possible. Pediatric GO patients tend to show comparable soft tissue inflammation (STS≥2); however, less motility dysfunction is defined as a decrease in 360° explants below ≤ 330° at the first clinical presentation compared with adult GO patients (*p* < 0,05). Although pediatric GO patients were reported more often with stages than adult patients were, proptosis, as previously defined, was noted as high in both cohorts (84% vs. 80%). On the other hand, lid retraction and Dysthyroid Optic Neuropathy (DON) were low in both cohorts (29% vs. 38 and 1% vs. 3%, respectively) (*p* > 0.05). The increased mild and less severe manifestations resulted in significantly fewer steroid treatments and interventions in pediatric than in adult GO patients (15% vs 42%, *p* < 0.001). Furthermore, adult patients were subjected significantly more often to orbital irradiation (only applied to patients > 35 years of age), as well as eye muscle and lid surgery and orbital decompression (Fig. 2 A, B).

## Thyroid management prior to first consultation

Pediatric GO patients were more likely to present with conservative medical therapy rather than definitive thyroid treatment prior to ophthalmological examination at our clinic. Thyroidectomy and the RAI were significantly greater in the adult cohort (10.8% vs 27.7% and 3.2% vs. 19.5%, respectively; *p* < 0.0005). ETA guidelines recommend avoiding RAI in children younger than 10 years and advise concurrent corticosteroid prophylaxis in older children if RAI is administered in the presence of inactive orbitopathy [17]. This explains the limited RAI use in our pediatric cohort. Medical anti-thyroid drugs regimens, on the other hand, were more likely to be effective in pediatric patients. Both medical therapy regimens block (44% vs. 25%) and block and replace (25.5% vs. 2%) were significantly more likely in the pediatric population (*p* < 0.0005) at initial presentation. Interestingly, 9/50 (18%) pediatric GO patients developed GO symptoms only after the initiation of ATD treatment. Finally, L-thyroxin supplementation was more common in the adult population (18.6% vs. 48.8%).

## Thyroid status and medical therapy at final presentation

While the number of thyroidectomized pediatric patients during the timespan of ophthalmological consultations at our department increased from 10.8% to 22.8% (by 12%) among pediatric patients, it increased by only 6.7% (from 27.7% to 34.4%) among adult GO patients. Accordingly, the number of RAI procedures increased by 2–3% in both cohorts during the same period.

### Thyroidectomy-induced TRAb negativity in pediatric patients with GO

Thyroidectomized patients had a median disease duration that was only 6 months longer than that of nonthyroidectomized patients before initial presentation at our department (Table 2).

**Table 2:** Duration of hypo-/hyperthyroidism before initial presentation

	Thyroidectomy	No-thyroidectomy
Number of Patients	20	61
Duration in months of hypo/hyperthyroidism manifestation before initial presentation	29.85±27* 18,26(1–90) ^1^	19,2 ±20* 12,03(1–87) ^1^

mean ±SD, ^1^Median (minimum - maximum)

**Fig. 3:**
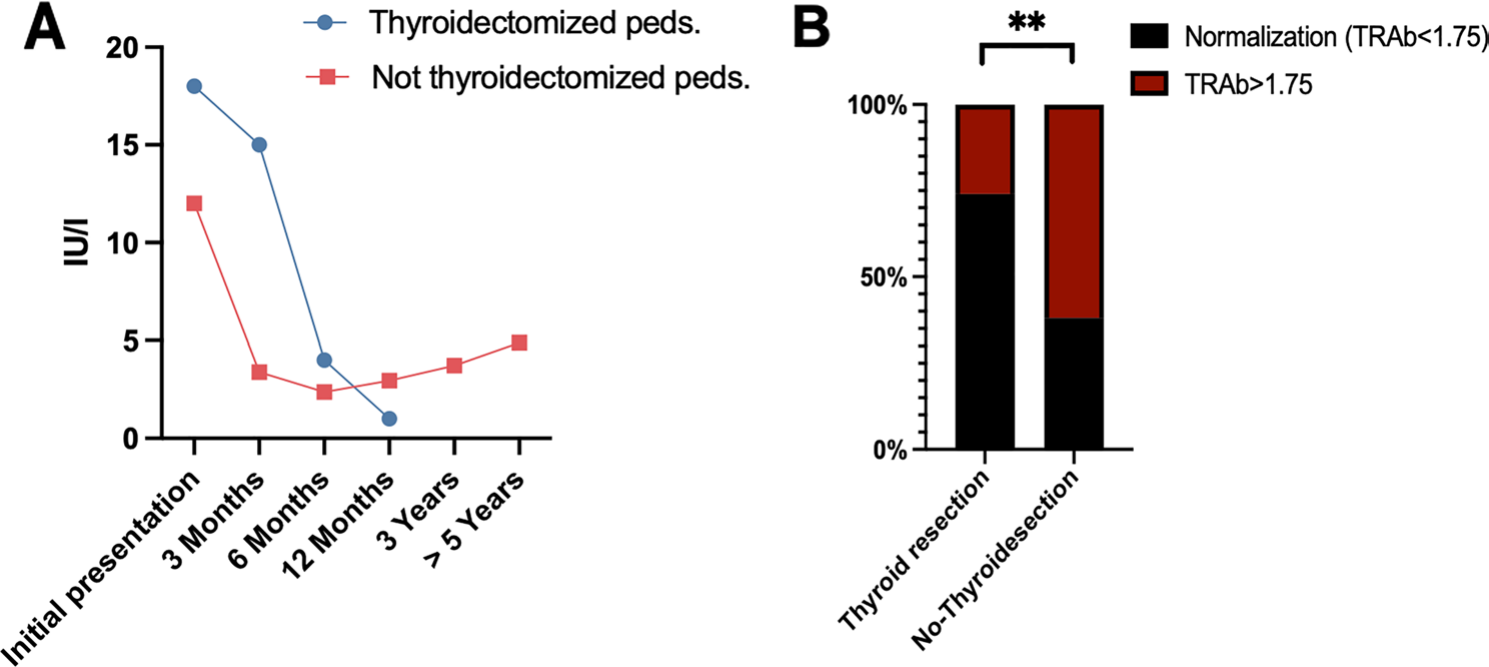
(**A**) scatter chart for median TRAb values (IU/L) in both subcohorts of pediatric GO patients: thyroidectomy vs. no thyroidectomy at observed time points. (**B**) percentage of pediatric GO patients with serological remission following thyroidectomy (TRAb < 1.75) within 40 months of observation

TRAb antibodies in two subcohorts (nontyroidectomized versus thyroidectomized GO patients) were assessed at different timepoints in pediatric GO patients. In thyroidectomized pediatric GO patients, the mean (± standard) preoperative TRAb (T1) (IU/l) was 25.9 (±21), 3–6 months postsurgery (T2) was 15.7 (±11), 6–12 months postsurgery (T3) was 4.9 (±4), and 12–35 months postsurgery (T4) was 2.2 (±3). In nonthyroidectomized pediatric GO patients, the mean (± SD) TRAb (IU/l) was 19.00 (±23) at first clinical presentation (T1), 11.40 (±19) at 3–6 months (T2), 6.86 (±10) at 6–12 months (T3), 7.88 (±16) at 12–35 months (T4), 3.58 (±1) at 35–50 months (T5) and 10.22 (±12) at 50–100 months (T6) (Fig. 3, A). TRAb normalization (< 1.75 IU/l) in the subgroup analysis of thyroidectomy vs no thyroidectomy patients revealed that thyroidectomized patients were significantly more often converted to normal patients (TRAb < 1,75: 73.9% vs. 38%) at the 40-month follow-up (Fig. 3, B).

### Early ATD-induced remission in nonthyroidectomized pediatric GO patients

**Fig. 4:**
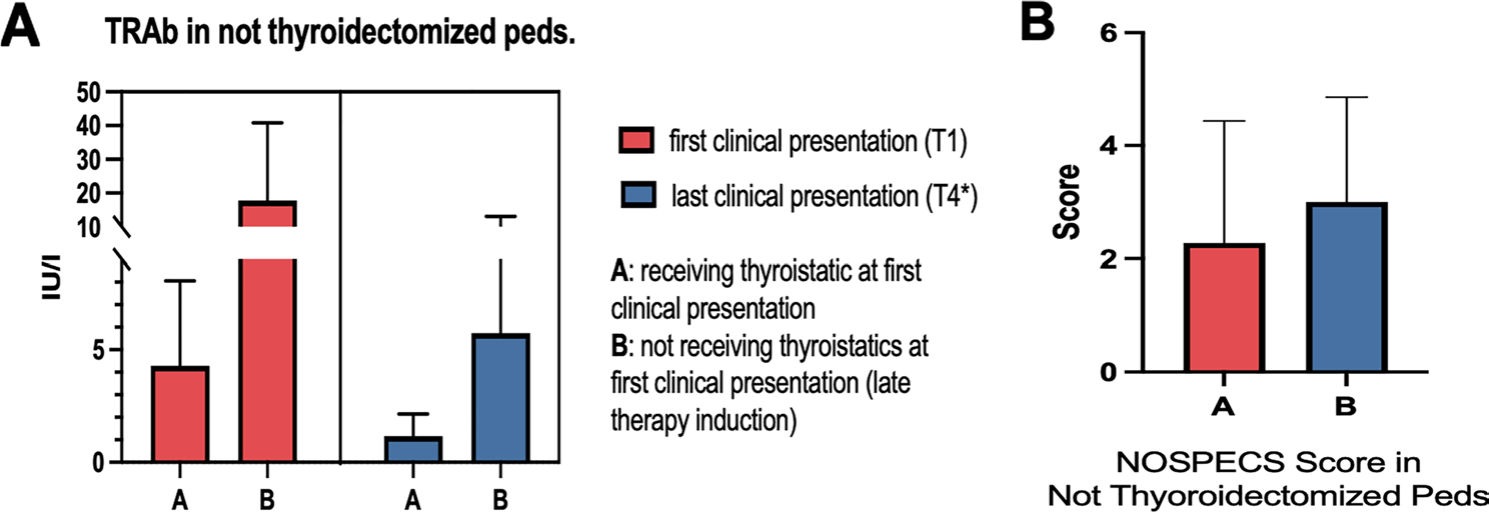
(**A**) bar graph for (TRAb) values in not thyroidectomized pediatric patients (Iu/l) receiving thyrostatic group a vs not-receiving thyrostatic therapy group B at first and last clinical presentation. (**B**) mean NOSPECS score in sub-cohort (group a vs group B) analysis of not thyroidectomized patients

Additionally, we compared the TRAb values of nonthyroidectomized children that were receiving ATD at first clinical presentation (A) at our department with those of children that were not receiving ATD initially at first and last clinical presentation (B). In nonthyroidectomized pediatric GO patients who received early ATD, the mean (± SD) TRAb at first clinical presentation (T1) (IU/l) was 4.3 (±4) and 1.2 (±1) at last follow-up > 40 months (T4). In nonthyroidectomized pediatric GO patients who delayed ATD treatment, the mean (± SD) TRAb (IU/l) was 17.8 (±23.1) at first clinical presentation (T1) and 5.7 (±7) at last follow-up > 40 months (T4*) (Fig. 3A). Two-way ANOVA revealed significant differences in TRAb values in the subgroup analysis of ATD-receiving patients (already receiving anti-thyroid drugs prior to consultation) vs non-ATD-receiving patients and anti-thyroid drugs induction patients after consultation at our eye department (*p* < 0.005) (Fig. 4A). However, the level of clinical activity (NOSPECS score) in group B was significantly greater (*p* < 0.0001) than that in group A (Fig. 4, B), which included pediatric GO patients (2.3 vs 3) (Table 3).

**Table 3:** Effect of late anti-thyroid drug initiation on the NOSPECS activity score

	A	B
Number of Patients	50	19
Duration in months of GO symptoms before presentation at our tertiary referral center	8.3(0–90)^1^	13.7(0–80)^1^
NOSPECS score* *p* < 0.0001^2^	2.3 ± 2	3±2

Unless otherwise stated, the data are presented as the means ± SDs, 1median (minimum - maximum), *NOSPECS was calculated as previously reported [24]. ^2^Wilcoxon signed rank test was used. Group A: ATD therapy induction prior to consultation at our department. Group B: ATD therapy induction during or after consultation at our department

### Influencing factors associated with remission in nonthyroidectomized pediatric GO patients

To further explore risk factors in nonthyroidectomized pediatric GO patients, univariate and multiple logistic regression analyses of different variables were conducted:

Univariate analyses revealed no significant influence of age at diagnosis, time of ocular symptoms or initial hormone concentrations on FT-4, TPO or anti-TG (Table 4). Among the evaluated variables, only FT3, TRAb, and TSH at initial presentation showed statistically significant associations with remission status in nonthyroidectomized pediatric GO patients, whereas all other parameters demonstrated no meaningful correlation.

**Table 4:** Univariate logistic regression analysis of factors associated with remission in a subcohort of nonthyroidectomized pediatric Graves’ disease patients

Variables (at first presentation)	Univariate-logistic Regression		
	OR	95% CI	p
Age	1.115	0.97–1.71	0.0754
FT-3	0.4197	0.17–0.72	0.0002*
FT-4	0.9452	0.86–1.01	0.2619
TPO	0,9154	0.99–1.00	0.9737
TRAb	0.9422	0.87–0.99	0.0049*
TSH	1.078	0.99–1.28	0.0343*
Anti-TG	0.9974	0.99–1.02	0.1123
Time from GD till GO	1.022	0.98–1.06	0.2061

**Fig. 5:**
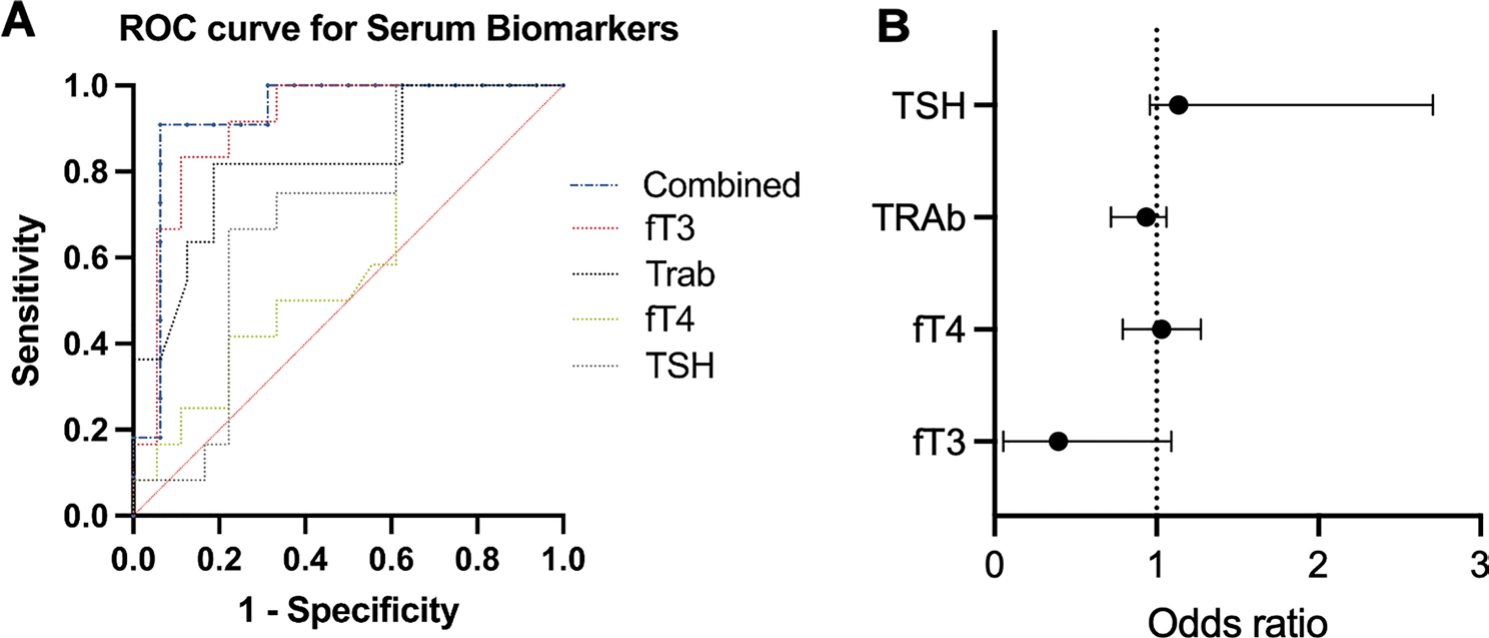
(**A**) ROC-Curve of multiple logistic regression to assess the diagnostic accuracy for likelihood of remission (serological remission TRAb < 1.75 with a mean follow-up of 53 (40–99) months in non-thyroidectomized pediatric GO-Patients. Serum biomarkers for fT3, fT4, TSH and TRAb at first clinical presentation was implemented. (**B**) Odd’s ratio plot of the multiple logistic regression, plotting the correlation between the variable of remission and the independent variables fT3, fT4, TRAb and TSH at first clinical manifestation

In addition, multiple logistic regression was performed with the dependent variable remission and the following independent (see multicollinearity test supplemental Table S1) variables: FT-3, FT-4, TRAb and TSH at first clinical manifestation. The combination of the parameters resulted in an AUC of 0.93 (*p=*0.0002, Tjur’s R squared = 0.57), a negative predictive power of 93.3% and a positive predictive power of 83.3% (Fig. 5 A, B).

## Discussion

Our large monocentric retrospective analysis demonstrated that pediatric patients with Graves’ orbitopathy (GO) exhibit distinct clinical and therapeutic characteristics from adult patients with GO. Furthermore, we were able to evaluate various treatment approaches, revealing that thyroidectomy could be considered more often in pediatric GO patients presenting with high initial TRAb levels since it results in rapid normalization of TRAb in a high proportion of pediatric patients.

### Patient characteristics

Our study confirms that proptosis is the most common clinical sign in pediatric patients with GO, followed by soft tissue inflammation and eyelid retraction, findings that are consistent with those of previous reports [17, 25]. Motility dysfunction was observed in 22% of our cohort, which is higher than the 11% reported by Durairaj et al. in 2006. Most pediatric patients presented with a mild form of GO (82%), similar to the rates reported in current European and Asian studies, such as [19, 26].

Compared with adults, pediatric GO patients are less likely to present with motility dysfunction, soft tissue involvement, or diplopia at their initial clinical visit. Moreover, pediatric patients more frequently present with mild disease, leading to a lower rate of corticosteroid use and surgical intervention. Notably, only a minority of the children (5/92) had received corticosteroids prior to their first consultation, whereas 43% of the adults had already been treated with corticosteroids at the time of referral. These discrepancies likely reflect both true differences in disease severity and limited experience among general pediatricians and ophthalmologists with pediatric GO, owing to its low prevalence. Importantly, our comparisons relied on objective ophthalmic parameters assessed at the first presentation, independent of prior corticosteroid exposure.

Consequently, pediatric GO should be managed in specialized centers and by experienced pediatric endocrinologists. Our findings suggest that autoimmune orbital disease in pediatric versus adult patients is a heterogeneous entity. Pediatric GO most commonly manifests during puberty, with a mean age of 12.3 ± 7 years—an essential period for both physiological and psychological development. Given the key role of thyroid hormones in growth and skeletal maturation during puberty, dysregulation may lead to unique pathophysiological consequences compared with those in adults.

### Thyroid treatment

Adult GO patients were more likely to have undergone definitive thyroid treatment before visiting our tertiary referral center—total thyroidectomy (27.7% vs. 10.8%) or radioactive iodine (RAI) therapy (19.5% vs. 3.2%). Unfortunately, definitive treatment does not mean that GO always calms rapidly. Radioiodine therapy is associated with a small but significant risk for new onset or deterioration of GO [27–29]; consequently, having undergone radioiodine therapy is also a significant risk factor for more severe stages of GO [30]. Thyroidectomy leads to a continuous decay of TRAb [28], but the size of the thyroid remnant matters. Subtotal thyroidectomy can exacerbate GO [31], indicating that residual thyroid tissue is dependent on TRAb and other autoantibody production. Similarly, Marroci et al. [32] demonstrated that incomplete thyroidectomy may lead to persistent ophthalmopathy requiring additional treatments such as orbital radiotherapy or high-dose glucocorticoids.

Surprisingly, pediatric GO patients were more frequently managed with conservative antithyroid drug (ATD) therapy prior to referral despite very high TRAb levels. To improve clinical decision-making, we compared outcomes between surgically and conservatively treated pediatric patients. The current Japanese [33] and European [17] guidelines recommend prolonged first-line ATD treatment (3–5 years or more), whereas the American Thyroid Association suggests considering definitive therapy if remission is not achieved within two years of ATD use [34]. This difference in approach is partly due to the increased risk of surgical complications in children (e.g., hypoparathyroidism and resultant hypocalcemia), underscoring the importance of high-volume surgical centers. [35, 36] Our data suggest significant benefits of thyroidectomy in patients with high TRAb values. Pediatric patients who underwent surgery had higher baseline TRAb levels but demonstrated a more rapid decline and higher rates of serological remission (TRAb < 1.75 UI: 74% vs. 38%) than did those managed conservatively.An important clinical question is whether the more rapid normalization of TRAb after thyroidectomy is preferable to a strategy of prolonged ATD therapy aiming at drug-free remission. Our data show that total thyroidectomy in pediatric GO is associated with faster and more frequent TRAb normalization compared with continued medical treatment. However, this does not necessarily imply that surgery is superior to prolonged ATD therapy in terms of long-term clinical or psychosocial outcomes. Thyroidectomy offers the advantages of rapid and stable biochemical control, absence of ATD-related adverse events, and potentially less prolonged autoimmune stimulation, but it also carries non-negligible surgical risks (e.g. hypoparathyroidism, recurrent laryngeal nerve injury) and entails lifelong levothyroxine replacement. In contrast, extended ATD therapy avoids surgery and may allow a subset of children to achieve permanent remission without ongoing medication, at the cost of variable relapse rates, possible serious side effects, and the burden of long-term treatment and monitoring.

Another subgroup comparison of our cohort revealed the effects of early referral and ATD treatment. We compared GO patients with and without ATD treatment at the first referral. In both groups, GO symptoms occurred at approximately the same time. However, the pediatric GO patients who had received ATD much earlier had lower TRAb and slightly milder GO than did the pediatric GO patients who did not.

### Factors influencing remission in pediatric Graves’ hyperthyroidism patients

The following parameters were included in the regression analysis to assess which factors influence the probability of remission: age and first visit: FT-3, FT-4, TPO Ab, TRAb, TSH, anti-TG Ab and time from GD until GO. Importantly, the timing of the first visit to our tertiary referral center varied with respect to the duration of preceding antithyroid drug (ATD) therapy. Nevertheless, we identified FT-3 and TRAb values at initial presentation as significant predictors of remission in the nonsurgical treated pediatric cohort. Elevated TRAb levels and high FT-3 concentrations were associated with poor disease control and a reduced likelihood of remission. For adults, Graves’ hyperthyroidism predictive scores, which include both TRAb and FT-3/FT-4 levels at the beginning of antithyroid drug therapy, have been established to predict the relapse/remission of hyperthyroidism. However, these scores, e.g., the Clinical Severity Score [37] (CSS) and Graves’ Recurrent Events After Therapy [38] (GREAT), are useful, although imperfect, tools for predicting the baseline relapse of hyperthyroidism. The areas under the curve (0,6 CSS − 0,63 (GREAT)) [39] were reached. The AUC reached for pediatric hyperthyroidism was 0.93. Owing to the small patient count, we should not consider small numbers too much here. These preliminary findings underscore the need for prospective studies to evaluate whether remission or relapse can be accurately predicted via longitudinal measurements of FT-3/FT-4, TRAb, age, and potentially additional biomarkers. The application of machine learning or artificial intelligence models may be particularly advantageous when incorporating sequential data points. In adult patients, serial measurements of TRAb levels during ATD therapy have shown improved predictive value, with the AUC increasing in relation to treatment duration [40–42]. In our cohort, higher initial FT3 and TRAb levels were associated with a lower likelihood of remission in non-thyroidectomized patients. This decrease is in line with the finding that follicular infiltration in thyroidectomized thyroids is correlated with TRAb levels [43]. While this finding may indicate that these biomarkers could help identify children at risk for a more persistent disease course, definitive conclusions regarding treatment strategies require prospective, comparative studies with standardized management protocols. Additionally, advancements in surgical techniques, such as the intraoperative use of indocyanine green (ICG) fluorescence imaging to identify parathyroid glands, have demonstrated potential in reducing the incidence of postoperative hypocalcemia [44]. A conceptual parallel may be drawn to thymectomy in myasthenia gravis, where prospective evidence has shown that surgical intervention significantly improves clinical outcomes, particularly among younger patients with recent disease onset [45]. This analogy underscores the broader principle that timely surgical intervention in autoimmune conditions can alter disease trajectory and improve patient quality of life.

### Limitations

This study is limited by its retrospective design. Nevertheless, it represents the largest monocentric pediatric GO cohort reported to date, and our findings are largely consistent with the literature. Future prospective, multicenter studies are needed to further elucidate the impact of thyroid treatment modalities in pediatric patients with GO.

## Conclusion

An analysis of the largest monocentric cohort of pediatric patients with Graves’ orbitopathy (pediatric-GO) confirmed that children usually develop a milder form of the disease, with proliferative processes predominating over inflammatory processes, as proptosis is the leading symptom. Consequently, children receive steroid therapy far less frequently. Whether new therapies targeting the TSHR/IGF-1 R pathway are effective in children—something that can be hypothesized—still needs to be investigated. However, in children, blocking antibodies that interfere with growth signals are certainly excluded.

Particularly interesting are the results of the regression analysis regarding factors influencing remission of hyperthyroidism pointing out a robust estimation of remission.

## Electronic supplementary material

Below is the link to the electronic supplementary material.

Supplementary Material 1

## Data Availability

The data that support the findings of this study are available from the corresponding author, K.A., upon reasonable request.
