# Supplementary material for: Pediatric Graves’ orbitopathy: TRAb and FT-3 are also prognostic factors in children—A tertiary center study
Source: Thyroid Res. 2026 Jan 22;19:5. doi: 10.1186/s13044-026-00286-7 (PMC12828925; doi:10.1186/s13044-026-00286-7)
Supplement: Supplementary file 1 — Supplementary Material 1 [file 13044_2026_286_MOESM1_ESM.docx]

**Supplemental Table 1: Multicollinearity testing:**

| Variables (at first presentation) | *VIF* | *R^2^ with other variables* |
| --- | --- | --- |
| FT-3 | 1.87 | 0.46 |
| FT-4 | 1.92 | 0.47 |
| TRAb | 1.45 | 0.31 |
| TSH | 1.08 | 0.08 |
